# Mini Review of Phytochemicals and Plant Taxa with Activity as Microbial Biofilm and Quorum Sensing Inhibitors

**DOI:** 10.3390/molecules21010029

**Published:** 2015-12-26

**Authors:** Chieu Anh Kim Ta, John Thor Arnason

**Affiliations:** Phytochemistry Laboratory, Department of Biology, University of Ottawa, Ottawa, ON K1N 6N5, Canada; kta074@uottawa.ca

**Keywords:** biofilm inhibitors, quorum sensing inhibitors, terpenes, quinones, organosulfur compounds

## Abstract

Microbial biofilms readily form on many surfaces in nature including plant surfaces. In order to coordinate the formation of these biofilms, microorganisms use a cell-to-cell communication system called quorum sensing (QS). As formation of biofilms on vascular plants may not be advantageous to the hosts, plants have developed inhibitors to interfere with these processes. In this mini review, research papers published on plant-derived molecules that have microbial biofilm or quorum sensing inhibition are reviewed with the objectives of determining the biosynthetic classes of active compounds, their biological activity in assays, and their families of occurrence and range. The main findings are the identification of plant phenolics, including benzoates, phenyl propanoids, stilbenes, flavonoids, gallotannins, proanthocyanidins and coumarins as important inhibitors with both activities. Some terpenes including monoterpenes, sesquiterpenes, diterpenes and triterpenes also have anti-QS and anti-biofilm activities. Relatively few alkaloids were reported. Quinones and organosulfur compounds, especially from garlic, were also active. A common feature is the polar nature of these compounds. Phytochemicals with these activities are widespread in Angiosperms in temperate and tropical regions, but gymnosperms, bryophytes and pteridophytes were not represented.

## 1. Introduction

Microbial biofilms are organized aggregations of cells attached to a substratum and surrounded by a self-produced extrapolymeric substance (EPS) matrix. These biofilms develop on many biotic and abiotic surfaces such as plant leaves and medical devices. The formation of a biofilm is a complex process, which requires the coordinated expression of many specific genes. The interconversion between planktonic and biofilm growth is controlled by a cell-to-cell communication system known as quorum sensing (QS). QS involves signal molecules called autoinducers that are released by the microbes themselves. Gram-positive bacteria use autoinducing peptides (AIPs) for signaling and Gram-negative bacteria have lipid-based molecules known as *N*-acyl-homoserine lactones (AHLs) [1,2,3,4,5]. In fungi such as *Candida albicans*, the quorum sensing molecules are farnesol and tyrosol [6,7]. Once populations reach a specific density or threshold, expression of certain genes such as virulence factors and adhesion proteins can occur. In many bacterial species, QS induction is required in order for biofilm formation to occur; in other species such as *Staphylococcus aureus*, repression of QS is necessary [8].

Many human pathogens are also biofilm formers; these are responsible for many persistent nosocomial infections especially in the immunocompromised population. These pathogens include *Pseudomonas aeruginosa* [9], *Burkholderia cepacia* [10], *Listeria monocytogenes* [11], *Staphylococcus aureus* [8] and *Candida albicans* [12], to name a few. With the rapid increase in antibiotic resistance to conventional therapies and the development of multi-resistant superbugs, the need for alternative antimicrobials is of particular interest. Current strategies to combat the increasing antibiotic resistance include targeting QS to prevent the formation of new biofilms and inhibiting the growth of existing biofilms. A recent comprehensive review [13] describes many chemical agents that inhibit bacterial biofilm formation derived from medicinal chemistry and biodiversity sources.

Within this large field of study, we focused in the present mini-review on the presence of quorum sensing and biofilm inhibitors against both bacteria and fungi in terrestrial plants, which has been documented in numerous plant species using various model microorganism bioassays. The presence of biofilms in plants is of interest for a fundamental understanding of the phenomenon in nature and also for its practical application. First, the discovery of these substances in plants informs chemical ecologists of potential new defense mechanisms in plants for future studies as well as which taxa and habitats are involved. In addition, it provides new insight into the use of these substances and plants in traditional medicine. The identification of the classes of plant derived secondary metabolites (phytochemicals) provides information on the evolution of these compounds, substances of interest for mechanistic studies and lead metabolites for analoging, quantitative structure-activity studies and drug development.

The objective of this mini review was to describe phytochemicals from terrestrial plant taxa and that can affect microbial quorum sensing and biofilm formation. Plant literature published before 2015 is summarized from searches that were performed using PubMed and Web of Science. The results are organized into a discussion of model organism bioassays used for assessing QS and biofilm inhibition, the different biosynthetic classes of active phytochemicals with representative substances and an overview of the taxa with activity.

## 2. Model Organism Bioassays

Bioluminescence or the production of light in marine bacteria such as *Vibrio harveyi* and *Vibrio fischeri* has been widely used to assess the ability of natural and synthetic compounds to interfere with quorum sensing in Gram-negative bacteria. In these species, light production is mediated by the *luxCDABEGH* operon, which is under QS control [14,15]. Similarly, *Chromobacterium violaceum* produces a purple pigment, violacein, which is also cell density-dependent. This species is popular in screening studies as it can be used to visually assess QS inhibition in a disc diffusion assay [16]. The *lux* operon is used in many reporter strains along with the *lacZ* gene and green fluorescent protein (GFP). These strains include *Agrobacterium tumefaciens* NTL4 [16], *Escherichia coli* MT102 (pSB403) [17], *Pseudomonas aeruginosa* PAO1 [18], and *Vibrio harveyi* [19], among others. The production of autoinducers can be quantified directly using luminescence, fluorescence, and absorbance of pigments or directly via chromatographic techniques such as High Performance Liquid Chromatography-Diode Array Detection (HPLC-DAD). For Gram-positive bacteria, QS inhibition is usually measured directly by quantifying the concentration of autoinducing peptides using HPLC coupled with mass spectrometry (MS) [20].

Many biofilm models have been developed using various microbial species and surfaces. The simplest and most popular assay involves growing a biofilm in a microtiter plate in the presence and absence of the compound of interest and then measuring the stained biofilm mass at the optimal wavelength [21]. This method can be used with fungi, Gram-positive and Gram-negative bacteria. In addition, dual-species and mixed-species biofilms has been studied with many different substrates.

## 3. Phytochemicals as QS and Biofilm Inhibitors

Phytochemicals reported in the literature are arranged according to the biosynthetic groups described in Dewick [22]. A discussion of their bioactivities follows.

### 3.1. Phenolics

#### 3.1.1. Phenylpropenoids

By far, phenolics represent the highest number of active compounds reported in terms of their effects on quorum sensing and biofilm formation when compared to all other classes (Table 1). Eugenol, a phenylpropene present in many plants, has been shown to inhibit the production of QS-mediated violacein in *Chromobacterium violaceum* and virulence factors in *Pseudomonas aeruginosa* PAO1 by 32% to 56% at concentrations of 50 to 200 μM, respectively [23]. This compound was also effective against biofilms of *Pseudomonas aeruginosa*, *Listeria monocytogenes* and *Klebsiella pneumoniae* clinical isolates [23,24,25]. Cinnamaldehyde, another phenylpropene, was reported by Brackman *et al.* [26] to interfere with the AI-2-mediated QS system in *Vibrio* spp. (65% inhibition at 100 µM). In terms of biofilm formation, cinnamaldehyde was shown to be effective against both Gram-positive and Gram-negative bacteria such as *Listeria monocytogenes* [24], *Staphylococcus epidermidis* [27], and *Cronobacter sakazakii* [28]. In these studies, the active concentration ranged from 946 µM to 38 mM, which were reported to inhibit the formation of new and preformed biofilms as well as downregulate the expression of biofilm-associated genes [24,27,28].

#### 3.1.2. Benzoic Acid Derivatives

Benzoic acid derivatives such as vanillin and gallic acid showed mixed effects depending on the organism and concentration tested. In a study by Ponnusamy *et al.* [29], 250 μg/mL of vanillin inhibited QS in *Chromobacterium violaceum* and biofilm formation in *Aeromonas hydrophila.* The anti-biofilm activity of vanillin was also confirmed by Kappachery *et al.* [30] using different abiotic surfaces. At a concentration of 0.18 mg/mL, pre-treatment with vanillin reduced the biofilm formation of *Aeromonas hydrophila* on membrane filters by 90% [30]. In another study, at 200 μg/mL vanillin enhanced AHL production in *Escherichia coli* JDL271/pAL105 and biofilm formation in *Pseudomonas aeruginosa* PAO1 and *Agrobacterium tumefaciens* C58 by at least two-fold [31]. Similarly, gallic acid at 200 μg/mL had no effect on *P. aeruginosa* PA14 biofilms [32] but inhibited *P. aeruginosa* PAO1 biofilm formation by 30% [31] and enhanced *Staphylococcus epidermidis* biofilms by three-fold at a similar concentration [33]. At a much higher concentration of 1 mM, gallic acid was shown to inhibit *Eikenella corrodens* biofilm formation by 80% [34]. In terms of QS, Borges *et al.* [35] saw a 59% reduction in violacein production at 1 mg/mL using *Chromobacterium violaceum.*

Other benzoic acid derivatives have also been reported to have anti-QS and anti-biofilm effects. Ellagic acid at 36 μg/disc showed greater QS inhibition in *C. violaceum* when compared to the positive control *Delisea pulchra* (Greville) Montagne extract [32]. This activity was confirmed by Huber *et al.* [17] who observed QS inhibition in *E. coli* MT102 and *Pseudomonas putida* at concentrations of 40 μg/mL and 30 μg/mL, respectively. This compound was not effective against *P. aeruginosa* PA14 biofilms at 10 μg/mL [32]. In another study, ellagic acid inhibited biofilm formation in various *Streptococcus dysgalactiae* strains by 70% at 4 μg/mL [36]. At higher concentrations of 15 to 40 μg/mL, ellagic acid was inhibitory against *Escherichia coli*, *Burkholderia cepacia*, *Staphylococcus aureus*, and *Candida albicans* biofilms [37].

**Table 1:** Phenolics and derivatives affecting microbial quorum sensing (QS) and/or biofilm formation.

Active Constituents (Source Plants)	Biological Activities	Ref.
**Phenylpropenoids**		
**eugenol** (various species) 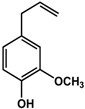	Inhibited *las* QS-mediated elastase production (32% at 200 μM) and *pqs* QS-mediated pyocanin production (56% at 50 μM) in *P. aeruginosa*, QS-mediated violacein production in *C. violaceum* (48% at 150 μM) Decreased biofilm formation in *P. aeruginosa* PAO1 (43% at 400 μM) Inhibited formation of new and inactivated preformed biofilms in *L. monocytogenes* (2.5 mM and 25 mM, respectively) Downregulated genes critical to *L. monocytogenes* biofilm formation (2.5 mM) Inhibited biofilm formation in *K. pneumoniae* clinical isolates (MIC = 63.5 μg/mL)	[23] [23] [24] [24] [25]
**cinnamaldehyde** (*Cinnamomum* sp.—Lauraceae) 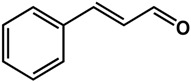	Inhibited formation of new and inactivated preformed biofilms in *L. monocytogenes* (0.75 mM and 10 mM, respectively) Inhibited AI-2-mediated QS in *Vibrio* spp. (65% at 100 μM) Downregulated genes involved in *L. monocytogenes* biofilm formation (0.75 mM) Inhibited biofilm formation in *S. epidermidis* (MIC = 125 μg/mL) Inhibited formation of new and inactivated mature biofilms in *C. sakazakii* (750 μM and 38 mM, respectively) and downregulated expression biofilm-related genes	[24] [26] [24] [27] [28]
**Benzoic Acid Derivatives**		
**vanillin** (*Vanilla planifolia* Jacks. ex Andrews—Orchidaceae) 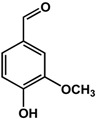	Inhibited violacein production in *C. violaceum* CV026 (69%) and biofilm formation in *A. hydrophila* (50%) at 0.25 mg/mL Inhibited biofilm formation in *A. hydrophila* on membrane filters by 90% with pre-treatment of 0.18 mg/mL Enhanced biofilm formation in *P. aeruginosa* PAO1 (3-fold at 200 μg/mL) and *A. tumefaciens* C58 (2-fold at 25 μg/mL) and AHL production in *E. coli* JDL271/pAL105 at 40 μg/mL	[29] [30] [31]
**gallic acid** (various species) 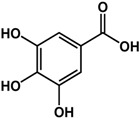	Enhanced biofilm formation in *P. aeruginosa* PAO1 (2-fold at 25 μg/mL) and *A. tumefaciens* C58 (2-fold at 100 μg/mL); reduced biofilm formation in *P. aeruginosa* PAO1 by 30% at 200 μg/mL Increased biofilm formation in *S. epidermidis* by 3-fold at 188 μg/mL Inhibited biofilm formation in *E. corrodens* by 80% at 1 mM Inhibited violacein production in *C. violaceum* ATCC 12472 (59% at 1 mg/mL)	[31] [33] [34] [35]
**ellagic acid** (various species) 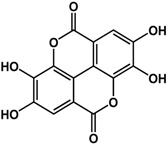	Inhibited biofilm formation (50% at 40 μg/mL) and swarming motility (100% at 20 μg/mL) in *B. cepacia* Inhibited QS in *E. coli* MT102 (pSB403) by 40% at 40 μg/mL and *P. putida* (pKR-C12) by 40% at 30 μg/mL Inhibited violacein production in *C. violaceum:* 18.3 mm at 36 μg Reduced biofilm formation in *S. dysgalactiae* strains by 70% at 4 μg/mL Inhibited biofilm formation in *S. aureus* ATCC 11632 (60% at 15 μg/mL), MRSA ATCC 33591 (70% at 20 μg/mL), *E. coli* ATCC 10536 (60% at 15 μg/mL), and *C. albicans* ATCC 90028 (50% at 20 μg/mL)	[17] [17] [32] [36] [37]
**Tannins**		
**1,2,3,4,6-penta-*O*-galloyl-ß-d-glucopyranose** (various species) 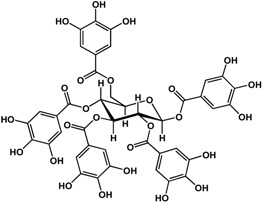	Inhibited biofilm formation in *S. aureus* (IC_50_ = 3.6 μM)	[38]
**punicalagin** (*Punica granatum* L. (Lythraceae) and Combretaceae species) 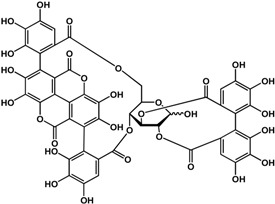	Inhibited violacein production in *C. violaceum* and swimming/swarming motility in *S. typhimurium* SL1344 at 15.6 μg/mL Downregulated expression of motility and QS related genes in *S. typhimurium* at 15.6 μg/mL	[39]
**hamamelitannin** (*Hamamelis virginiana* L.—Hamamelidaceae) 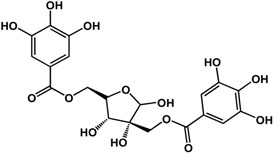	Inhibited *agr* QS regulator RNAIII and δ-hemolysin production at 50 μg/mL Reduced cell attachment of MRSA to polystyrene plate at 4 μg/mL In mice infection model, treatment of grafts with 30 mg/mL showed no detectable MRSA and MRSE load after 7 days	[40]
**tannic acid** (various species) 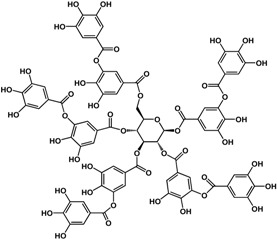	Enhanced biofilm formation in *P. aeruginosa* PA14 at 100 μg/mL Inhibited QS in *P. putida* (pKR-C12) by 40% at 30 μg/mL and *E. coli* MT102 (pSB403) by 20% at 60 μg/mL Inhibited violacein production in *C .violaceum* (21.8 mm at 500 μg) Inhibited biofilm formation in *P. aeruginosa* PA14 (72% at 200 μg/mL) Inhibited *S. aureus* biofilm formation (60% at 2 μM) and increased *isaA* expression (a transglycosylase) Reduced ex*pression o*f genes responsible for QS and virulence in *S. aureus* at 20 μg/mL Inhibited biofilm formation in *S. aureus* by >50% at 20 μg/mL	[17] [17] [32] [32] [41] [42] [42]
**Stilbenes**		
**resveratrol** (various species) 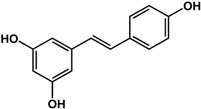	Inhibited *S. aureus* biofilm formation (30%) and enhanced *S. epidermidis* biofilm formation (1.5-fold) at 100 μg/mL Inhibited biofilm formation in *P. aeruginosa* PA14 and *E. coli* O157:H7 at 50 μg/mL Inhibited *P. acnes* biofilm formation by 80% at 0.32%	[33] [42] [43]
**pterostilbene** (*Vitis* sp.—Vitaceae and Ericaceae species) 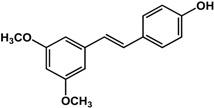	Inhibited formation of new and mature *C. albicans* SC5314, Y0109, 0304103, and 01010 biofilms at 16 μg/mL Inhibited hyphal formation in *C. albicans* at 4 μg/mL Treatment of 16 μg/mL altered expression of genes involved in morphological transition, ergosterol biosynthesis, filamentation, and cell surface proteins; also effective in rat central venous catheter infection model	[44]
**Flavonoids**		
**quercetin** (various species) 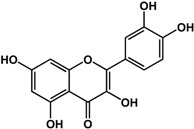	Inhibited AI-induced bioluminescence in *V. harveyi* BB886, MM32 (75% at 6.25 μg/mL) and biofilm formation in *E. coli* O157:H7, *V. harveyi* BB120 (60% at 6.25 μg/mL) Inhibited biofilm formation in MRSA (>80%) and MSSA (>50%) strains at 1 μg/mL Reduced expression of genes involved in QS and virulence in *S. aureus* at 10 μg/mL	[45] [46] [46]
**(−)-catechin** (*Camellia sinensis* (L.) Kuntze—Theaceae and others) 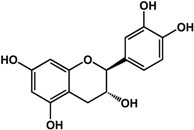	Inhibited violacein production in *C. violaceum* CV026 (50% at 2 mM), pyocyanin (50% at 0.25 mM) and elastase production (30% at 4 mM) in *P. aeruginosa* PAO1 Reduced biofilm formation and downregulated QS genes expression in *P. aeruginosa* PAO1 by at 4 mM	[47]
**(−)-epicatechin** (*Camellia sinensis* (L.) Kuntze—Theaceae and others) 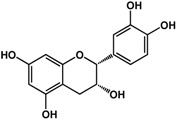	Increased AHL production in *E. coli* JDL271/pAL105 at 40 to 200 μg/mL Enhanced biofilm formation in *P. aeruginosa* PAO1 (2-fold at 200 μg/mL) and *A. tumefaciens* C58 (2-fold at 400 μg/mL) Inhibited biofilm formation in *E. coli* JM109 by 40% at 1 mg/mL Inhibited violacein production in *C. violaceum* ATCC 12472 (33% at 1 mg/mL) Inhibited elastase activity in *P. aeruginosa* PAO1 by 40% at 4 mM	[31] [31] [35] [35] [47]
**(–)-gallocatechin** (*Camellia sinensis* (L.) Kuntze—Theaceae) 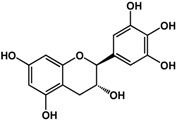	Inhibited biofilm formation in *E. corrodens* by >60% at 1 mM (MIC = 0.5 mM)	[34]
**(–)-epigallocatechin** (*Camellia sinensis* (L.) Kuntze—Theaceae and others) 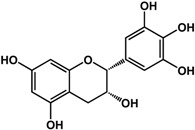	Inhibited biofilm formation in *E. corrodens* by >60% at 1 mM (MIC = 0.25 mM)	[34]
**(–)-catechin gallate** (*Camellia sinensis* (L.) Kuntze—Theaceae) 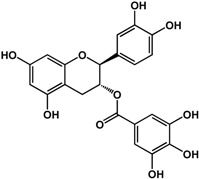	Inhibited biofilm formation in *E. corrodens* by >60% at 1 mM (MIC = 0.1 mM)	[34]
**(–)-epicatechin gallate** (*Camellia sinensis* (L.) Kuntze—Theaceae and others) 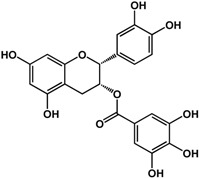	Inhibited biofilm formation in *E. corrodens* by >60% at 1 mM (MIC = 0.1 mM)	[34]
**(–)-gallocatechin gallate** (*Camellia sinensis* (L.) Kuntze—Theaceae) 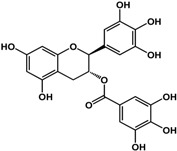	Inhibited biofilm formation in *E. corrodens* by >60% at 1 mM (MIC = 0.1 mM)	[34]
**(−)-epigallocatechin gallate** (*Camellia sinensis* (L.) Kuntze—Theaceae) 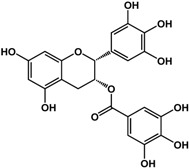	Inhibited QS in *E. coli* MT102 (pSB403) and *P. putida* (pKR-C12) (>50% and 40% at 40 μg/mL, respectively) Reduced biofilm formation (30%) and swarming motility in *B. cepacia* (100%) at 40 μg/mL Inhibited biofilm formation in *E. corrodens* by >60% at 1 mM (MIC = 0.1 mM)	[17] [17] [34]
**Diarylheptanoids**		
**curcumin** (*Curcuma longa* L.—Zingiberaceae) 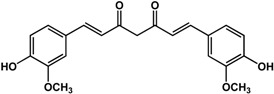	Inhibited biofilm formation in *S. epidermidis* (MIC = 25 μg/mL) Inhibited biofilm formation and AHLs production in *P. aeruginosa* at 1 μg/mL Altered expression of QS-related genes, reduced virulence factors production (60% to 80%) and mortality in infection models (28% to 80%) with treatment of 3 μg/mL in *P. aeruginosa* PAO1 Completely inhibited biofilm formation in *H. pylori* ATCC 43504 and other clinical isolates at 8 μg/mL for up to 10 days Inhibited sortase A activity (IC_50_ = 10 μM) and biofilm formation in *S. mutans* UA159 (at 15 μM) Completely eradicated 48-h and 14-day biofilms and reduced biomass of 8-week biofilm in *E. faecalis* ATCC 29212 (625 μg/mL) Inhibited biofilm formation (50%), alginate production (20% to 70%), and motility in *V. harveyi*, *V. parahaemolyticus*, and *V. vulnificus* at 75 μg/mL Inhibited violacein production in *C. violaceum* CV026 and virulence factors production in *P. aeruginosa* PAO1 and *S. marcescens* FJ584421 at 100 μg/mL (56%–63%) Inhibited swimming motility by 50% in *E. coli* ATCC 10536 (50 μg/mL), *P. aeruginosa* PAO1 (50 μg/mL), *P. mirabilis* ATCC 7002 (75 μg/mL), and *S. marcescens* FJ584421 (75 μg/mL) Inhibited biofilm formation in *E. coli* (52%), *P. aeruginosa* PAO1 (89%), *P. mirabilis* (52%), and *S. marcescens* (76%) at 100 μg/mL Inhibited biofilm formation (50%) and surface adhesion (15%) in *C. albicans* at 50 μg/mL	[27] [48] [48] [49] [50] [51] [52] [53] [53] [53] [54]

#### 3.1.3. Tannins

Tannins including ellagitannins and proanthocyanidins are another type of phenolic that has documented biofilm and quorum sensing inhibitory activities. At a concentration of ~4 μM, 1,2,3,4,6-penta-*O*-galloyl-β-d-glucopyranose (a common precursor of gallotannins) inhibited biofilm formation in *Staphylococcus aureus* by 50% [38]. Punicalagin [39] and hamamelitannin [40] are other tannins that can interfere with QS in Gram-negative and Gram-positive bacteria, respectively. In a study by Li *et al.* [39], punicalagin (an ellagitannin found in pomegranate and Combretaceae species) inhibited violacein production in *C. violaceum* as well as swimming and swarming motility in *Salmonella* *typhimurium* SL1344 at 15.6 μg/mL. Further analyses by these authors showed a downregulation of QS and motility-related genes in *S. typhimurium* at the same concentration. Similarly, hamamelitannin (a gallotannin from American witch-hazel) was shown to reduce cell attachment of methicillin-resistant *Stapylococcus aureus in vitro* at 4 μg/mL [40]. At a higher concentration of 50 μg/mL, δ–hemolysin production and QS regulator RNAIII in *S. aureus* were inhibited by hamamelitannin. Furthermore, this decrease in virulence was confirmed *in vivo* with a graft infection model using rats. At pre-treatment of 30 mg/mL, implanted grafts showed no detectable methicillin-resistant *Staphylococcus aureus* (MRSA) and methicillin-resistant *Staphylococcus epidermidis* (MRSE) loads after 7 days [40]. Tannic acid was reported to be active against both Gram-negative at Gram-positive bacteria. At 100 μg/mL, Huber *et al.* [17] showed an enhancement of biofilm formation in *Pseudomonas aeruginosa* PA14; however, a 72% inhibition was observed at 200 μg/mL against PA14 biofilms in another study [32]. In other studies, anti-biofilm activities were shown at lower concentrations from 3.4 to 20 μg/mL against *S. aureus* [41,42]. In terms of QS, inhibition was observed in *Pseudomonas putida* at 30 μg/mL [17] and *S. aureus* at 20 μg/mL [42].

#### 3.1.4. Stilbenes and Flavonoids

Stilbenes such as resveratrol and pterostilbene have been reported to interfere with the formation of both fungal and bacterial biofilms. In a study by Cho *et al.* [42], 50 μg/mL of resveratrol inhibited *P. aeruginosa* PA14 and *E. coli* O157:H7 biofilms. In another study, Coenye *et al.* [43] showed that resveratrol at 0.32% also inhibited biofilm formation in *Propionibacterium acnes*. At a higher concentration of 100 μg/mL, this compound was inhibitory to *S. aureus* biofilms but actually enhanced biofilm formation in *S. epidermidis* [33]. For pterostilbene, Li *et al.* [44] showed that treatment of 16 μg/mL inhibited new and mature biofilms in various *Candida albicans* strains. In the same study, at a concentration of 4 μg/mL pterostilbene prevented hyphal formation in the same fungal strains. Transcriptomic analyses showed that this compound altered the expression of genes involved in morphological transition, ergosterol biosynthesis, filamentation and cell surface proteins. Furthermore, in a rat central venous catheter infection model, treatment of pterostilbene showed anti-biofilm effects in a dose-dependent manner [44].

Flavonoids are another type of phenolic that showed inhibitory activities in quorum sensing and biofilm formation. In a study by Vikram *et al.* [45], quercetin was shown to inhibit bioluminescence in *Vibryo harveyi* strains by 75% at a concentration of 6.25 μg/mL. Lee *et al.* [46] reported anti-biofilm activities of this compound against *E. coli* O157:H7 and *V. harveyi* BB120 at the same concentration as well as inhibition of *S. aureus* biofilms at 1 μg/mL. Microarray analyses by the same authors showed that quercetin reduced the expression of genes involved in QS and virulence of *S. aureus.* Other flavonoids such as catechins from green tea (*Camellia sinensis* L.) also showed similar effects. In a study by Matsunaga *et al.* [34], (−)-gallocatechin, (−)-epigallocatechin, (−)-catechin gallate, (−)-epicatechin gallate, (−)-gallocatechin gallate, and (−)-epigallocatechin gallate all inhibited biofilm formation in *Eikenella corrodens* at 1 mM. (−)-catechin, a related compound, also inhibited violacein and virulence factors production in *Chromobacterium violaceum* and *Pseudomonas aeruginosa* PAO1, respectively [47]. However, (−)-epicatechin (another related compound) showed different activity depending on the tested organism. At 200 μg/mL, (−)-epicatechin enhanced biofilm formation in *P. aeruginosa* PAO1 and *Agrobacterium tumefaciens* C58 [31]. At higher concentrations of 1 mg/mL, this compound inhibited *Escherichia coli* JM109 biofilms by 40% [35]. In terms of QS, (−)-epicatechin inhibited violacein production in *C. violaceum* at 1 mg/L [35] but increased AHL production in *E. coli* DL271/pAL105 at concentrations of 40 to 200 μg/mL [31]. (−)-epigallocatechin gallate also showed QS inhibitory activities against *E. coli* MPT102 and *Pseudomonas putida* as well as swarming motility in *Burkholderia cepacia* at 40 μg/mL [17].

#### 3.1.5. Diarylheptanoids

Another noteworthy phenolic in terms of QS and biofilm activities is curcumin (a diarylheptanoid found in turmeric). This compound has been well studied and showed various pleiotropic biological effects. Karaman *et al.* [55] showed that this compound enhanced biofilm formation in *Staphylococcus aureus* at 16 μg/mL. The opposite effect was seen by Rudrappa and Bais [48] and Pattiyathanee *et al.* [49] where curcumin inhibited AHL production and biofilm growth in *P. aeruginosa* PAO1 at 1 μg/mL and completely inhibited biofilm formation in *Helicobacter pylori* clinical isolates at 8 μg/mL, respectively. At higher concentrations from 25 to 625 μg/mL, this compound displayed anti-biofilm activities against *Staphylococcus epidermidis, Vibrio* sp., *Escherichia coli, Pseudomonas aeruginosa* PAO1, *Proteus mirabilis*, *Serratia marcescens*, *Klebsiella pneumoniae*, *Enterococcus faecalis*, *Streptococcus mutans* and *Candida albicans* [25,27,50,51,52,53,54]. In terms of QS, curcumin inhibited violacein production in *C. violaceum* as well as virulence factors production in *Vibrio* sp., *P. aeruginosa* PAO1, *S. marcescens* at concentrations ranging from 3 μg/mL to 100 μg/mL [48,52,53].

### 3.2. Terpenoids

Different types of terpenes such as monoterpenes, limonoids, and triterpenes have also been reported to have anti-biofilm and/or anti-QS activities (Table 2). Thymol and carvacrol (monoterpenes) were shown to be effective against new and existing biofilms of Gram-positive and Gram-negative bacteria [24,56,57]. In a study by Upadhyay *et al.* [24], thymol inhibited the formation of new *Listeria monocytogenes* biofilms and inactivated preformed ones at 0.5 mM and 5 mM, respectively. At the lower concentration, genes critical to *L. monocytogenes* biofilm development were downregulated [24]. Using the same model organism, the authors showed that carvacrol also effective at concentrations of 0.65 mM and 10 mM against new and existing biofilms, respectively. This compound also downregulated biofilm-associated genes in *L. monocytogenes* at 0.65 mM [24]. In another study, Soumya *et al.* [56] reported inhibitory activities of these monoterpenes against different strains of *Pseudomonas aeruginosa.* At a concentration of 0.1%, thymol reduced the biofilm mass of *P. aeruginosa* strains ATCC 27853, CIP A22 and IL5 by 86%, 54% and 70%, respectively. Similarly, 0.04% of carvacrol inhibited biofilms of the same strains by more than 90% [56]. The inhibitory activity of thymol was also confirmed by Qiu *et al.* [57] where treatment with 64 µg/mL resulted in more than five-fold reduction of enterotoxin genes expression in *Staphylococcus aureus*.

Sesquiterpenoids such as salvipisone and acanthospermolides were shown to reduce biofilm growth in *P. aeruginosa, S. aureus,* and *Staphylococcus epidermidis* [58,59,60]. At a concentration of 37.5 µg/mL, salvipisone (from the roots of *Salvia sclarea* L.) inhibited preformed *S. epidermidis* biofilms by at least 85% and preformed *S. aureus* biofilms by 85% [58,59]. In another study, a number of acanthospermolides isolated from *Acanthospermum hispidum* DC. showed inhibitory activities against *P. aeruginosa* biofilms by at least 70% at 2.5 µg/mL [60].

**Table 2:** Terpenoids affecting microbial quorum sensing (QS) and/or biofilm formation.

Active Constituents (Source Plants)	Biological Activities	Ref.
**Monoterpenes**		
**thymol** (*Thymus vulgaris* L.—Lamiaceae) 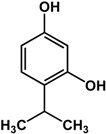	Inhibited formation of new and inactivated preformed biofilms in *L. monocytogenes* (0.5 mM and 5 mM, respectively) Downregulated genes critical to *L. monocytogenes* biofilm formation (0.5 mM) Inhibited formation of biofilm in *P. aeruginosa* ATCC 27853, CIP A22, and IL5 at 0.1% by 86%, 54%, and 70%, respectively Downregulated expression of enterotoxin genes in *S. aureus* at 64 μg/mL (>5-fold)	[24] [24] [56] [57]
**carvacrol** (*Thymus vulgaris* L. and other Lamiaceae species) 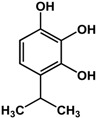	Inhibited formation of new and inactivated preformed biofilms in *L. monocytogenes* (0.65 mM and 10 mM, respectively) Downregulated genes critical to *L. monocytogenes* biofilm formation (0.65 mM) Inhibited formation of biofilm in *P. aeruginosa* ATCC 27853, CIP A22, and IL5 at 0.04% by >90%	[24] [24] [56]
**Sesquiterpenes**		
**salvipisone** (*Salvia sclarea* L.—Lamiaceae) 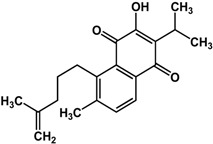	Reduced preformed biofilm of *S. epidermidis* RP12 (>90%), 6756/99 (85%) and *S. aureus* 1474/01 (85%) at 37.5 μg/mL	[58,59]
**acanthospermolide** (*Acanthospermum hispidum* DC.—Asteraceae) 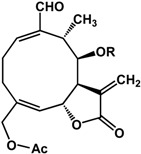	Inhibited biofilm formation in *P. aeruginosa* (70% at 2.5 μg/mL)	[60]
**Triterpenoids**		
**isolimonic acid** (*Citrus* × *aurantium* L.—Rutaceae) 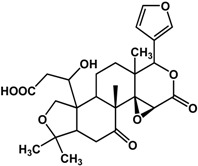	Inhibited AI-induced bioluminescence in *V. harveyi* BB170 (60% at 6.25 μg/mL) and biofilm formation in *V. harveyi* BB120 (40% at 100 μg/mL)	[61]
**ichangin** (*Citrus* × *aurantium* L.—Rutaceae) 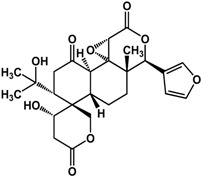	Inhibited AI-induced bioluminescence in *V. harveyi* BB170 (90%) and biofilm formation in *V. harveyi* BB120 (40%) at 100 μg/mL	[61]
**betulinic acid** (various species) 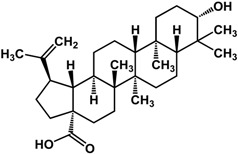	Enhanced biofilm formation in *P. aeruginosa* PA14 at 100 μg/mL	[42]
**ursolic acid** (various species) 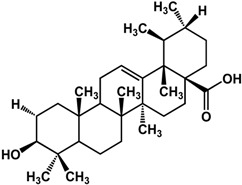	Inhibited biofilm formation in *P. aeruginosa* PAO1 (>87%), *E. coli* JM109 (50%), and *V. harveyi* BB120 (57%) at 10 μg/mL Induced expression of chemotaxic and motility genes and repressed sulphur metabolism in *E. coli* K-12 at 10 μg/mL Inhibited biofilm formation in *P. aeruginosa* PAO1 by 35% at 10 μg/mL	[21] [21] [62]
**gymnemic acids** (*Gymnema sylvestre* (Retz.) R.Br. ex Sm.—Apocynaceae and Asclepiadaceae species) 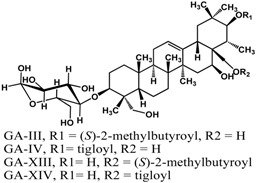	Mixture of 4 acids at 40 μg/mL inhibited yeast-to-hypha transition in *C. albicans* SC5314 and induced conversion of hyphae back to yeast form (100% after 11 h) Inhibited conidial germination and hyphal growth in *Aspergillus fumigates* by 74% at 40 μg/mL Treatment of 40 μg/mL mixture improved survival in *C. albicans*-fed *C. elegans* infection model (100% rescue) by inhibiting formation of invasive hyphae	[63]

Limonoids (derived from triterpenes) isolated from *Citrus* × *aurantium* L. (bitter orange) showed anti-QS activities at low concentrations [61]. These include isolimonic acid, ichangin and several other compounds; a 17% to 83% inhibition of bioluminescence in *Vibryo harveyi* was observed at 6.25 μg/mL and dose-dependent activity was seen for all compounds tested [61]. Other triterpenoids are reported to have differing activities. Betulinic acid, a lupane triterpene, showed no activity at 5 μg/mL in a bioassay with *P. aeruginosa* PA14 [32]; at 100 μg/mL biofilm formation is greatly enhanced in the same strain [42]. Ursane triterpenoids, on the other hand, have been documented to have good inhibitory activities. Ursolic acid has been shown to inhibit *P. aeruginosa* PAO1, *E. coli* JM109, and *V. harveyi* BB120 biofilms by 35% to 87% at 10 μg/mL [21,62]. This compound was not active against *P. aeruginosa* PA14 biofilm at a concentration of 5 μg/mL [32]. Furthermore, gymnemic acids (ursane triterpene glycosides) isolated from *Gymnema sylvestre* (Retz.) R.Br. ex Sm. (Apocynaceae) have been reported by Vediyappan *et al.* [63] to inhibit yeast-to-hypha transition in *Candida albicans* SC5314. At 40 µg/mL, the four-compound mixture inhibited hyphal transition and induced the conversion of hyphae to yeast form; 100% conversion was observed after 11 h. These compounds also inhibited conidial germination and hyphal growth in *Aspergillus fumigates* at the same concentration. Furthermore, treatment of the gymnemic acids mixture, at 40 μg/mL, significantly improved the survival rate of *Candida albicans*-infected *Caenorhabditis elegans* and resulted in 100% rescue [63].

### 3.3. Sulfur-Containing Phytochemicals

Sulfur-containing compounds (Table 3) such as allicin, ajoene, and thiocyanates have been shown to interfere with biofilm formation and quorum sensing of both Gram-positive and Gram-negative bacteria [32,64,65,66,67,68]. Allicin (from garlic) inhibited *Staphylococcus epidermidis* and *Pseudomonas aeruginosa* PAO1 biofilm adhesion as well as the expression of QS-regulated virulence factors in *P. aeruginosa* PAO1 [64,65]. At a concentration of 1.1 mg/mL, a 74% reduction in biofilm mass was observed in *P. aeruginosa* PA14 [32]. In another study using GFP-transformed *P. aeruginosa* PAO1, treatment of 128 µg/mL of allicin resulted in a 50% decrease in biofilm thickness and a 70% decrease of EPS production [64]. For *S. epidermidis* strains, 4 mg/mL of allicin inhibited biofilm formation by more than 90% [65]. Similarly, ajoene (also from garlic) inhibited QS in reporter strains *P. aeruginosa* lasB-gfp, *P. aeruginosa* rhlA-gfp and *E. coli* luxI-gfp with 50% effective concentrations (EC_50_) ranging from 15 to 100 µM [66]. In the same study, ajoene also downregulated virulence factors expression (elastase, rhamnolipid, enterotoxins) by five-fold at 80 µg/mL [66]. Furthermore, using a mouse pulmonary infection model, subcutaneous treatment of ajoene at 25 mg/kg, significantly improved bacteria clearance after three days [66]. Thiocyanates such as sulforaphane and allyl isothiocyanate (from Brassicaceae species) have been reported to inhibit QS in *Escherichia coli* and *Chromobacterium violaceum* as well as reduce biofilm formation in *Pseudomonas aeruginosa* and *Listeria monocytogenes* [35,67,68]. In a study by Ganin *et al.* [67], complete inhibition of QS in *E. coli* DH5 (pJN105L) (pSC11) was observed at 100 µM of sulforaphane. This compound also inhibited biofilm formation and pyocyanin production (QS-mediated) in *P. aeruginosa* PAO1 by 60% and 70% at concentrations of 37 µM and 100 µM, respectively. Similarly, allyl isothiocyanate was shown to reduce violacein production (QS-controlled) in *C. violaceum* by 70% at 5 µg/mL [66]. The anti-biofilm activity of this compound was demonstrated in *L. monocytogenes*, *P. aeruginosa* and *E. coli* where treatment with 1 mg/mL resulted in 61 to 100% inhibition [68].

**Table 3:** Other biosynthetic classes of phytochemicals affecting microbial quorum sensing (QS) and/or biofilm formation.

Active Constituents (Source Plants)	Biological Activities	Ref.
**Sulfur-Containing Compounds**		
**allicin** (*Allium sativum* L.—Amaryllidaceae) 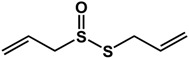	Inhibited *P. aeruginosa* PA14 biofilm (74% at 1 μL). Reduced adhesion of GFP-transformed *P. aeruginosa* PAO1, EPS production (70%), biofilm thickness (50%), and expression of virulence factors at 128 μg/mL Inhibited biofilm formation by >90% in *S. epidermidis* strains at 4 mg/mL	[32] [64] [65]
**ajoene** (*Allium sativum* L.—Amaryllidaceae) 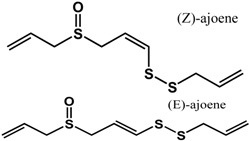	Inhibited QS in *P. aeruginosa lasB-gfp* (IC_50_ = 15 μM)*, rhlA-gfp* (IC_50_ = 50 μM)*, E. coli luxI-gfp* (IC_50_ = 100 μM) reporter strains Downregulated QS-regulated virulence factors (5-fold at 80 μg/mL) and improved bacteria clearance in mouse infection model (subcutaneous treatment of 25 mg/kg body weight)	[66] [66]
**sulforaphane** (Brassicaceae species) 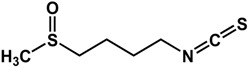	Inhibited *lasR*-mediated QS in *E. coli* DH5 (pJN105L) (pSC11) completely at 100 μM Inhibited biofilm formation (60% at 37 μM) and pyocyanin production in *P. aeruginosa* PAO1 (70% at 100 μM)	[67]
**Sulfur-Containing Compounds**		
**allyl isothiocyanate** (Brassicaceae species) 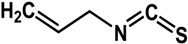	Inhibited violacein production in *C. violaceum* ATCC 12472 (70% at 5 μg/mL) Inhibited biofilm formation in *L. monocytogenes* (61%), *P. aeruginosa* (90%), *E. coli* (100%) at 1 mg/mL	[35] [68]
**Coumarins**		
**aesculetin** (various species) 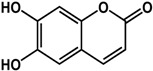	Inhibited QS in *C. violaceum* CV026, *P. aeruginosa, E. coli* JB523 (30% to 60% at 500 μM) Inhibited biofilm formation in *S. aureus* strains (53% to 77% at 128 μg/mL) Reduced expression of biofilm-related genes (motility, adhesion, virulence) in *E. coli* O157:H7 at 50 μg/mL	[18] [36] [69]
**umbelliferone** (Apiaceae species) 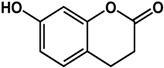	Inhibited biofilm formation in *E. coli* O157:H7 (>80% inhibition at 50 μg/mL) Reduced expression of biofilm-related genes (motility and adhesion) in *E. coli* O157:H7 at 50 μg/mL Inhibited formation of *S. aureus* CECT 976 biofilm by 50% at 800 μg/mL	[69] [69] [70]
**Quninones**		
**chrysophanol** (various species) 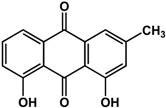	Inhibited biofilm formation in *P. aeruginosa* PAO1 (44%) and *S. maltophilia* (38%) at 200 μM	[71]
**emodin** (various species) 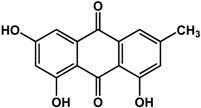	Inhibited biofilm formation in *P. aeruginosa* PAO1 (75%) and *S. maltophilia* (43%) at 20 μM	[71]
**shikonin** (Boraginaceae species) 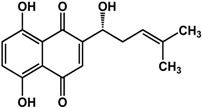	Inhibited biofilm formation in *P. aeruginosa* PAO1 (44%) and *S. maltophilia* (54%) at 200 μM	[71]
**purpurin** (*Rubia tinctorum* L.—Rubiaceae) 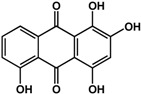	Inhibited yeast-to-hypha transition in *C. albicans* SC5314 at 3 μg/mL Inhibited formation of new and preformed *C. albicans* biofilms (50% at 5 μg/mL and 30% at 10 μg/mL, respectively) Downregulated expression of hypha-specific genes in *C. albicans* at 5 to10 μg/mL	[72]
**Alkaloids**		
**berberine** (Berberidaceae species and others) 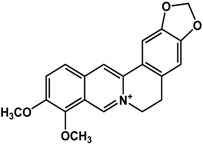	Inhibited biofilm formation in *K. pneumoniae* clinical isolates (MIC = 63.5 μg/mL) Inhibited biofilm formation in *S. epidermidis* ATCC 35984 (50% at 30 μg/mL) and SE243 (50% at 45 μg/mL)	[25] [73]
**chelerythrine** (Papaveraceae species) 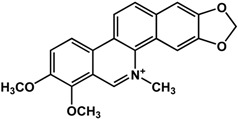	Inhibited biofilm formation in *S. aureus* 6538P (50% at 15 μM) and *S. epidermidis* RP62A (50% at 9 μM)	[74]
**sanguinarine** (Papaveraceae species) 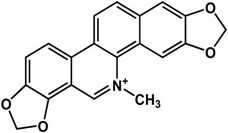	Inhibited biofilm formation in *S. aureus* 6538P (50% at 25 μM) and *S. epidermidis* RP62A (50% at 5 μM)	[74]
**Alkaloids**		
**reserpine** (*Rauwolfia* sp.—Apocynaceae) 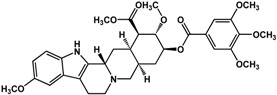	Inhibited biofilm formation in *K. pneumoniae* clinical isolates (MIC = 15.6 μg/mL)	[25]

### 3.4. Coumarins

Coumarins have been documented to possess QS and biofilm inhibitory activities (Table 3). Aesculetin was reported to inhibit QS in *C. violaceum, P. aeruginosa,* and *E. coli* JB523 by 30% to 78% at 500 μM [18]. In other studies, Dürig *et al.* [36] and Lee *et al.* [69] showed that aesculetin was effective against *S. aureus* biofilms (>50% inhibition at 128 μg/mL) and reduced the expression of biofilm-related genes (at 50 μg/mL) in *E. coli* O157:H7, respectively. Furthermore, aesculetin decreased Shiga-like toxin production in *E. coli* O157:H7 and reduced virulence in a *C. elegans* infection model [69]. Lee *et al.* [69] also reported that umbelliferone (another coumarin) inhibited biofilm formation (90%) and expression of motility and adhesion genes expression in *E. coli* O157:H7 at a concentration of 50 µg/mL. The anti-biofilm activity of umbelliferone was also confirmed in *S. aureus* CECT976 by Monte *et al.* [70] where treatment with 800 µg/mL caused a 50% inhibition in biofilm formation.

### 3.5. Quinones

Quinones have also been reported to have anti-biofilm activities against bacterial and fungal species (Table 3). In a study by Ding *et al.* [71], quinones such as chrysophanol, emodin, and shikonin all showed inhibitory activities against biofilms of *Pseudomonas aeruginosa* PAO1 and *Stenotrophomonas maltophilia*. Emodin was more 10 times more active than both chrysophanol and shikonin as treatment with 20 µM caused a 75% reduction in biofilm growth for *P. aeruginosa* PAO1 and a 43% reduction in *S. maltophilia.* Chrysophanol and shikonin both required a higher concentration of 200 µM to elicit the same level of activity in both bacterial species. Purpurin, another quinone, was shown to repress yeast-to-hypha transition in *Candida albicans* SC5314 at 3 μg/mL [72]. At higher concentrations of 5 to 10 μg/mL, this compound was effective against new and existing *C. albicans* biofilms (30% to 50% inhibition). Further analyses showed that purpurin downregulated the expression of hypha-specific genes [72].

### 3.6. Alkaloids

In regard to alkaloids, relatively few compounds have been reported to have inhibitory activities against bacterial biofilm (Table 3). In particular, Wang *et al.* [73] and Magesh *et al.* [25] showed that berberine inhibited the biofilm formation in *Staphylococcus eipidermidis* and *Klebsiella pneumoniae*, respectively. At a concentration of 63.5 μg/mL, berberine decreased biofilm growth in different *K. pneumoniae* clinical isolates [25]. Treatment of 30 to 45 µg/mL of berberine resulted in 50% inhibition of *S. epidermidis* biofilms [73]. Similarly, chelerythrine and sanguinarine were also effective against Gram-positive biofilms of *S. aureus* and *S. epidermidis* at micromolar concentrations [74]; the 50% inhibitory concentrations were reported to range from 15 to 25 µM for *S. aureus* and 5 to 9 µM for *S. epidermidis* [74] Resperine, an alkaloid from *Rauwolfia* sp. (Apocynaceae), also showed inhibitory activity against *K. pneumoniae* biofilms with a minimum inhibitory concentration of 15.6 μg/mL [25].

Overall the literature shows that the biosynthetic classes of compounds inhibiting biofilms and QS are diverse. The published literature to date reports activity mostly with phenolics, terpenoids, organosulfur compounds, and quinones but relatively few alkaloids or other types of secondary metabolites reported.

## 4. Taxa and Habitats

A wide variety of plant families are represented in the literature from which active compounds were isolated. At least 21 families are described in Table 1, Table 2 and Table 3. They are angiosperms and many more could be added if the re-occurrence of compounds in different families is considered. However, this represents a relatively small number of the total 642 plant families described in The Plant List [75]. Both monocots and eudicots are represented but gymnosperms, bryophytes and pteridophytes are either absent or under-represented in the literature. Many plant families are characteristic of tropical or subtropical areas such as Zingiberaceae, Rubiaceae, Lauraceae and Theaceae or temperate areas such as Asteraceae, Lamiaceae, Ericaceae, Berberidaceae and Apiaceae. Few records describe plant families of arid areas or dry habitats. For example, there are no records of common families Cactaceae, Poaceae, or Crassulaceae. This may be a sampling bias and there are few systematic studies to make any firm conclusions at this time but this could be a rich area of study. Our own observations are that tropical plants are a rich source of biofilm and QS inhibitors [76]. In rainforests, the high humidity and ever-wet conditions are ideal for bacterial biofilm growth. When these occur on leaves, the exopolysaccharide layer is a perfect habitat for germination of bryophyte spores, which leads to fouling of leaves and a decline in plant health as fouled leaves cannot performance photosynthesis (the bryophyte mat prevents sunlight from reaching the mesophyll layer).

## 5. Conclusions

The results presented in this mini review show that plant species contain many reported phytochemicals that interfere with microbial quorum sensing and biofilm formation. The biosynthetic classes of compounds involved is diverse but has not been systematically examined. The identified inhibitors may be a rich mine of lead compounds to produce drugs to address the growing problem of antibiotic resistance or to develop adjuvant therapies to impede pathogen success. The chemical ecology of these compounds as natural defenses is still largely untouched and their role in traditional medicines is an interesting topic for future investigation.

## Abbreviations

AHL: *N*-acyl-homoserine lactone
AIPs: autoinducing peptides for signaling
EPS: extrapolymeric substance
GFP: green fluorescent protein
HPLC-DAD: High performance liquid chromatography, diode array detection
QS: quorum sensing
